# Supplementary material for: The Cutaneous leishmaniasis impact questionnaire: Translation, cross-cultural adaptation and validation in adults with Cutaneous leishmaniasis in Ethiopia
Source: PLoS Negl Trop Dis. 2026 Mar 13;20(3):e0014073. doi: 10.1371/journal.pntd.0014073 (PMC12987440; doi:10.1371/journal.pntd.0014073)
Supplement: S1 Table — (DOCX) [file pntd.0014073.s001.docx]

| ***Additional file 1****:*  Expert scores and agreement to measure relevance of CLIQ items for CL patients (N=6) from ALERT and Boru Meda Hospital, 2023. | | | | | | | | | | | | |
| --- | --- | --- | --- | --- | --- | --- | --- | --- | --- | --- | --- | --- |
| **የቆዳ ቁንጭር አጠቃላይ ተጽዕኖ**  *_General impact of Cutaneous Leishmaniasis_* | *Expert scores* | | | | | | | | |  | | |
|  | *Exp 1* | *Exp 2* | *Exp 3* | *Exp 4* | *Exp 5* | *Exp 6* | *Exp agreed* | *I-CVI* | *UA* | *Pc* | *K** | *Decision* |
| 1. የቆዳ ቁንጭር በአጠቃላይ ጤንነትዎ ላይ ምን ተፅኖ አሳድሯል?   *_How does cutaneous leishmaniasis affected your overall health?_* | *4* | *4* | *4* | *4* | *3* | *4* | *6* | *1* | *1* | *0.0156* | *1.0* | *perfect* |
| 1. የቆዳ ቁንጭር በአካላዊ እንቅስቃሴዎት ላይ ምን ያህል እንቅፋት ፈጥሯል?   _How much does cutaneous leishmaniasis interfered with your physical activity?_ | *4* | *4* | *4* | *4* | *1* | *3* | *5* | *0.83* | *0* | *0.09375* | *0.81* | *perfect* |
| 1. የቆዳ ቁንጭር የመሥራት ችሎታዎት/ትምህርቶት ላይ በሆነ መንገድ ምን ያህል ተፅኖ አሳድሯል?   _How much has cutaneous leishmaniasis affected your job skills/studies?_ | *4* | *4* | *3* | *4* | *4* | *4* | *6* | *1* | *1* | *0.0156* | *1.0* | *perfect* |
| 1. የቆዳ ቁንጭር በሆነ መንገድ የህክምና ወጪዎን ምን ያህል ጨምሯል?   _How much has cutaneous leishmaniasis increased your medical expenses?_ | *4* | *3* | *3* | *4* | *4* | *4* | *6* | *1* | *1* | *0.0156* | *1.0* | *perfect* |
| 1. የቆዳ ቁንጭር የቤተሰብዎን የገንዘብ በጀት ምን ያህል ጎድቶታል ብለው ያስባሉ?   _How much damage do you think cutaneous leishmaniasis has caused to your family's financial budget?_ | *3* | *3* | *3* | *4* | *4* | *4* | *6* | *1* | *1* | *0.0156* | *1.0* | *perfect* |
| 1. የቆዳ ቁንጭር ከያዞት ወዲህ ከሌሎች እንደ ተነጠሉ ይሰማዎታል?   _How isolated have you felt since you had cutaneous leishmaniasis?_ | *4* | *4* | *4* | *3* | *4* | *3* | *6* | *1* | *1* | *0.0156* | *1.0* | *perfect* |
| 1. የእርሶ ቆዳ በቆዳቸው ላይ ቁስል ከሌሉባቸው ሰዎች ጋር ሲነፃፀር የተለየ ነው ብለው በማሰብ ምን ያህል ተጨንቀው ያውቃሉ?   *_How often have you suffered thinking that your appearance is different from people who don´t have wounds on their skin?_* | *4* | *4* | *3* | *4* | *4* | *3* | *6* | *1* | *1* | *0.0156* | *1.0* | *perfect* |
|  |  |  |  |  |  |  |  |  |  |  |  |  |
| **ኣካላዊ፣ ስነ-ልቦናዊ፣ ማህበራዊና ኢኮኖሚያዊ ተጽእኖ**  *_Physical, psychological, social and economic impact_* |  |  |  |  |  |  |  |  |  |  |  |  |
| 1. በቆዳዎት ላይ ባሉ ቁስሎች ምክንያት በእግር መራመድ ፣ ልብስ መልበስ ወይ መታጠብ ተቸግረው ያውቃሉ?   *Have you had difficulty walking, changing clothes or bathing because of the wound (s) on your skin?* | *4* | *4* | *4* | *3* | *1* | *2* | *4* | *0.67* | *0* | *0.2344* | *0.5* | *Moderate* |
| 1. በቆዳው ቁስል (ሎች) ቦታ ላይ ህመም ፣ ማቃጠል ፣ ማሳከክ ወይም አለመመቸት ተሰምቶት ያውቃል?   *_Have you felt pain, burning, itching or discomfort at the site of the skin wound (s)?_* | *4* | *4* | *4* | *4* | *2* | *3* | *5* | *0.83* | *0* | *0.09375* | *0.81* | *perfect* |
| 1. በቆዳ ቁንጭር ምክንያት ጭንቀት፣ ሀዘን ወይም ፍርሃት ተሰምቶት ያውቃል?   _Have you ever felt nervous, sad or scared because of Cutaneous Leishmaniasis?_ | *4* | *4* | *4* | *4* | *4* | *4* | *6* | *1* | *1* | *0.0156* | *1.0* | *perfect* |
| 1. በቆዳ ቁንጭር ምክንያት የጥፋተኛነት ወይም በራስ መተማመን የማጣት ስሜት ተሰምቶት ያውቃል?   *_Have you ever felt guilty or insecure about Cutaneous Leishmaniasis?_* | *4* | *4* | *3* | *4* | *4* | *3* | *6* | *1* | *1* | *0.0156* | *1.0* | *perfect* |
| 1. *በቆዳ ቁስል (ቁስሎ) ምክንያት ሀፍረት* ተሰምቶት ያውቃል?   *Have you ever felt embarrassed because of the skin wound(s)?* | *4* | *4* | *3* | *3* | *4* | *3* | *6* | *1* | *1* | *0.0156* | *1.0* | *perfect* |
| 1. በቆዳ ቁንጭር በሽታ ምክንያት ከስራ ገበታዎ /ከትምህርት ቤት ቀርተው ያውቃሉ?   Have you ever missed work (or school) because of Cutaneous Leishmaniasis? | *4* | *4* | *3* | *4* | *2* | *4* | *5* | *0.83* | *0* | *0.09375* | *0.81* | *perfect* |
| 1. በቆዳ ቁስለት (ቁስሎች) ምክንያት በግብረ ሥጋ ግንኙነት ጊዜ ችግር አጋጥሞዎት ያውቃል?   Have you ever had difficulty during sexual intercourse because of the skin wound(s)? | *4* | *4* | *3* | *3* | *2* | *1* | *4* | *0.67* | *0* | *0.2344* | *0.5* | *Moderate* |
| 1. እርሶ ወደ ጤና አገልግሎት ተቋም በሚመጡበት ግዜ የእርሶና የቤት እና የስራ ደርሻ ሸፍኖ ለሚሰራ ሰው ምን ያህል ጊዜ መክፈል ነበረብዎት?   How often have you had to pay someone to replace you in work or home activities so you could go get health service? | *4* | *3* | *2* | *4* | *3* | *2* | *4* | *0.67* | *0* | *0.2344* | *0.5* | *Moderate* |
| 1. ሌሎች ሰዎች በቆዳ ቁስለት ላይ ባላቸው ጥሩ ያልሆነ አመለካከት ምክንያት የአለባበስ ዘይቤዎን (ልብስ፤ ሻርፕ፡መነፅር፡ኮፍያ) ቀይረዋል?   Did you have to change the style of dressing because of other people's prejudices about their skin wounds? | *4* | *4* | *4* | *4* | *3* | *3* | *6* | *1* | *1* | *0.0156* | *1.0* | *Perfect* |
| 1. በቆዳ ቁንጭር ምክንያት ማህበራዊ እንቅስቃሴዎች ላይ ከመሳተፍ ምን ያህል ተቆጥበዋል?   How often have you avoided social activities with groups of people because of Cutaneous Leishmaniasis? | *4* | *4* | *4* | *4* | *3* | *4* | *6* | *1* | *1* | *0.0156* | *1.0* | *Perfect* |
| 1. የቆዳ ቁንጭርን ለመታከም ወደ ሕክምና ቀጠሮዎ በሚመጡበት ወቅት ምን ያህል የሎሎች ሰዎች እርዳታ ያስፈልጎታል?   How often do you depend on someone else to accompany you to your medical appointments to treat Cutaneous Leishmaniasis? | *4* | *3* | *2* | *3* | *2* | *3* | *4* | *0.67* | *0* | *0.2344* |  |  |
| **ስለ ጤና አገልግሎቶች እና ህክምና ግንዛቤ**  **Perception about health services and treatment** |  |  |  |  |  |  |  |  |  |  |  |  |
| 1. የቆዳ ቁንጭርን ለማከም ይወስዱት ስለነበረው መድሃኒት ምን ያስባሉ?   What do you think about the medication you used to treat Cutaneous Leishmaniasis? | *4* | *4* | *4* | *4* | *3* | *3* | *6* | *1* | *1* | *0.0156* | *1.0* | *Perfect* |
| 1. የቆዳ ቁንጭርን ምርመራ ፈልገዉ ወደ ጤና አገልግሎቶች በመጡበት ወቅት ስለተደረገሎት አቀባበል ምን ያስባሉ?   What did you think about how you were welcomed by the health services when you were seeking diagnosis of Cutaneous Leishmaniasis? | *3* | *4* | *4* | *4* | *3* | *3* | *6* | *1* | *1* | *0.0156* | *1.0* | *Perfect* |
| 1. የቆዳ ቁንጭርን መድሃኒት ፈልገዉ ወደ ጤና አገልግሎቶች በመጡበት ወቅት ስለተደረገሎት አቀባበል ምን ያስባሉ?   What did you think about how you were welcomed by the health services when you were seeeking treatment for Cutaneous Leishmaniasis? | *3* | *4* | *4* | *3* | *3* | *4* | *6* | *1* | *1* | *0.0156* | *1.0* | *Perfect* |
|  |  |  |  |  |  |  |  |  |  |  |  |  |
| 1. ከቆዳ ቁንጭር ለመዳን በወሰዱት መድኃኒቶች ምክንያት ምን ያህል ጊዜ ህመም ተሰምቶዎታል?   How often have you felt sick because of the medications you took to treat Cutaneous Leishmaniasis? | *4* | *3* | *4* | *4* | *3* | *3* | *6* | *1* | *1* | *0.0156* | *1.0* | *Perfect* |
| 1. አቅርቦቶችን እንዲያቀርቡልዎ ወይም የቁስሉን ማሸጊያ እንዲለውጥሎት ምን ያህል በህክምና አገልግሎቶች ላይ ተማምነዋል?   How often have you relied on health services to provide you with supplies or to help changing the wound bandages? | *4* | *2* | *3* | *2* | *1* | *1* | *2* | *0.33* | *0* | *0.2344* | *0.15* | *Slight* |
|  |  |  |  |  |  |  |  |  |  |  |  |  |
| 1. የቆዳ ቁንጭር ህክምናን ለማግኘት የሚደረግ ጥረት ምን ያህል አስፈላጊ ነው ብለው ያስባሉ?   How much do you care about the need to seek health services for the treatment of cutaneous leishmaniasis? | *4* | *4* | *3* | *4* | *3* | *3* | *6* | *1* | *1* | *0.0156* | *1.0* | *Perfect* |
| 1. የቆዳ ቁንጭር ጋር በተያያዘ ምርመራዎችን ለማካሄድ ፣ የሕክምና ቀጠሮዎች ወይም ሆስፒታል ለመተኛት ምን ያህል ጊዜ ወስዷል?   How long has it taken to get the tests done, medical appointments or hospitalizations related to Cutaneous Leishmaniasis? | *4* | *4* | *4* | *4* | *3* | *3* | *6* | *1* | *1* | *0.0156* | *1.0* | *Perfect* |
| Proportion Relevance  S-CVI based on Ave proportion Relevance = 0.9 | *1* | *0.96* | *0.92* | *0.96* | *0.72* | *0.84* |  | *S-CVI Ave=*  *0.9* | *S-CVI UA Ave= 0.68* |  |  |  |
