# Supplementary material for: The Cutaneous leishmaniasis impact questionnaire: Translation, cross-cultural adaptation and validation in adults with Cutaneous leishmaniasis in Ethiopia
Source: PLoS Negl Trop Dis. 2026 Mar 13;20(3):e0014073. doi: 10.1371/journal.pntd.0014073 (PMC12987440; doi:10.1371/journal.pntd.0014073)
Supplement: S2 Table — (DOCX) [file pntd.0014073.s002.docx]

**S2 Table**: Confirmatory factor analysis results with factor loading, Construct reliability and Average variance extracted for patient with CL

| **Factors** | **Items** | Factor loading | Error variance | CR | AVE |
| --- | --- | --- | --- | --- | --- |
| General Impact | G1-Impact on Overall well being | 0.67 | 0.0 | 0.98 | 0.57 |
|  | PF4-Difficulty on *walking,* changing clothes or bathing | 0.51 | -1.03 |  |  |
|  | PF5-Feeling pain, burning, itching or discomfort at the site of the skin wound(s) | 0.61 | -0.10 |  |  |
|  | PF6-Impact on Physical activities | 0.60 | 0.27 |  |  |
|  | PF7-Difficulty during sexual intercourse | 0.33 | 0.34 |  |  |
|  | E8-Feeling embarrassed | 0.83 | 0.18 |  |  |
|  | E9-Feeling nervous, sad or scared | 0.78 | -0.11 |  |  |
|  | E10-Suffered due to thinking that owns appearance is different from people who don´t have wounds on their skin | 0.75 | -0.07 |  |  |
|  | E11-Feeling guilty or insecure | 0.75 | -0.88 |  |  |
|  | O12-ever missed work (or school) | 0.66 | -0.92 |  |  |
|  | O13-affected once ability to work (or study) | 0.69 | 0.56 |  |  |
|  | Ec14-increased Once health expenses | 0.69 | 0.53 |  |  |
|  | Ec15-financially damaged you family´s budget | 0.59 | 1.49 |  |  |
|  | Ec16-had to pay someone to replace you in work or home activities so you could go get health service | 0.53 | -0.65 |  |  |
|  | S17-change the style of dressing because of other people's prejudices | 0.58 | 0.67 |  |  |
|  | S18-avoided social activities with groups of people | 0.66 | -1.07 |  |  |
|  | S19-feel isolated from others | 0.80 | 0.97 |  |  |
| Treatment and Health service satisfaction | TI20-What do you think about the medication you used to treat C.L? | 0.61 | -1.47 | 0.78 | 0.34 |
|  | TI21-felt sick because of the medications took to treat CL | 0.70 | -0.17 |  |  |
|  | TI22-care about the need to seek health services for the treatment of CL | 0.32 | 0.61 |  |  |
|  | HSS23-Perception about ways welcomed by the health services while seeking diagnosis for CL | 0.38 | -0.26 |  |  |
|  | HSS24-Perception about ways welcomed by the health services while seeking treatment for CL? | 0.53 | -1.64 |  |  |
|  | HSS25-Perception on time it takes to get the tests done, medical appointments or hospitalizations related to CL | 0.28 | 1.57 |  |  |
|  | HSS26-Dependency on someone else to accompany you to your medical appointments to treat C. L. | 0.19 | 5.19 |  |  |
|  | HSS27-Relied on health services to provide you with supplies or to help changing the wound bandages | 0.41 | -0.62 |  |  |

CL-Cutaneous Leishmaniasis, CR- Construct reliability, AVE-Average variance explained
