# Supplementary material for: The Cutaneous leishmaniasis impact questionnaire: Translation, cross-cultural adaptation and validation in adults with Cutaneous leishmaniasis in Ethiopia
Source: PLoS Negl Trop Dis. 2026 Mar 13;20(3):e0014073. doi: 10.1371/journal.pntd.0014073 (PMC12987440; doi:10.1371/journal.pntd.0014073)
Supplement: S3 Table — (PDF) [file pntd.0014073.s003.pdf]

የቆዳ ሊሽማኒያሲስ *(ቁንጭር)* ተጽዕኖ መጠይቅ (CLIQ)

የመረጃ ቅጽ መለያ ቁጥር፡______________________________

የመረጃ ሰበሳቢው ስም፡ ______________________________

መረጃው የተበሰበበት ቀን__________/ወር_____/____ዓ.ም

ከዚህ በታች ያሉት ጥያቄዎች በህይወትህ/ሽ ውስጥ የቆዳ ሊሽማኒያሲስ/ቁንጭር/ሻህን (CL) ያደረሰውን ተፅእኖን ይገመግማሉ።

ለእያንዳንዱ ለሚከተሉት ጥያቄዎች እባክዎን ከዚህ በፊት በበሽታው ምክንያት የደረሰቦትን ተፅኖ በተሻለ ሁኔታ የሚገልጽ መልስን ይስጡ። ለእያንዳንዱ ጥያቄ መልስ ለመስጠት ሕመምዎት ከመጀመሪያ እስከ አሁን ያለዉን ሁኔታ ከግምት ውስጥ ያስገቡ ።

	**የቆዳ ሊሽማኒያሲስ አጠቃላይ ተጽዕኖ**	*ኣይመለከተኝም*	*ምንም*	*በጥቂቱ*	በመጠኑ	መሀከለኛ	*ከፍተኛ*
1.	የቆዳ ሊሽማኒያሲስ *(ቁንጭር)* በአጠቃላይ ጤንነትዎ ላይ ምን ያህል ተፅኖ አሳድሯል?	0	0	1	2	3	4
2.	የቆዳ ሊሽማኒያሲስ *(ቁንጭር)* በአካላዊ እንቅስቃሴዎት ላይ ምን ያህል እንቅፋት ፈጥሯል?	0	0	1	2	3	4
3.	የቆዳ ሊሽማኒያሲስ *(ቁንጭር)* የመሥራት ችሎታዎት/ትምህርቶት ላይ ምን ያህል ተፅኖ አሳድሯል?	0	0	1	2	3	4
4.	የቆዳ ሊሽማኒያሲስ *(ቁንጭር)* የህክምና ወጪዎን ምን ያህል ጨምሯል?	0	0	1	2	3	4
5.	የቆዳ ሊሽማኒያሲስ *(ቁንጭር)* የቤተሰብዎን የገንዘብ በጀት ላይ ምን ያህል ጉዳት አድርሷል ብለው ያስባሉ?	0	0	1	2	3	4
6.	የቆዳ ሊሽማኒያሲስ ከያዞት ወዲህ ከሌሎች እንደ ተነጠሉ ምን ያህል ይሰማዎታል?	0	0	1	2	3	4
7.	የእርሶ ቆዳ በቆዳቸው ላይ ቁስል ከሌሉባቸው ሰዎች ጋር ሲነፃፀር የተለየ ነው ብለው በማሰብ ምን ያህል ተጨንቀው ያውቃሉ?	0	0	1	2	3	4
	**አካላዊ፥ ስነ-ልቦናዊ፥ ማህበራዊ እና ኢኮኖሚያዊ ተጽዕኖ**	*ኣይመለከተኝም*	በጭራሽ	*ከስንት አንድ ግዜ*	አንዳንድ ጊዜ	ብዙ ጊዜ	*በጣም ብዙ ግዜ*
8.	በቆዳዎት ላይ ባሉ ቁስሎች ምክንያት በእግር ለመራመድ፣ ልብስ ለመልበስ ወይም ለመታጠብ ምን ያህል ጊዜ ተቸግረው ያውቃሉ?	0	0	1	2	3	4
9.	በቆዳዎ ቁስል (ሎች) ቦታ ላይ ህመም፣ ማቃጠል፣ ማሳከክ ወይም አለመመቸት ምነ ያህል ጊዜ ተሰምትዎት ያውቃል?	0	0	1	2	3	4
10.	በቆዳዎት ላይ ባሉ ቁስሎች ምክንያት በእግር ለመራመድ፣ ልብስ ለመልበስ ወይም ለመታጠብ ምን ያህል ጊዜ ተቸግረው ያውቃሉ?	0	0	1	2	3	4

11.	በቆዳ ሊሽማኒያሲስ *(ቁንጭር)* ምክንያት የጥፋተኛነት ወይም በራስ መተማመን የማጣት ስሜት ምን ያህል ጊዜ ተሰምቶት ያውቃል?	0	0	1	2	3	4
12.	*በቆዳዎት ቁስል ምክንያት ምን ያህል ጊዜ ሀፍረት* ተሰምቶት ያውቃል?	0	0	1	2	3	4
13.	በቆዳ ሊሽማኒያሲስ *(ቁንጭር)* በሽታ ምክንያት ከስራ ገበታዎ /ከትምህርት ቤት ምን ያህል ጊዜ ቀርተው ያውቃሉ?	0	0	1	2	3	4
14.	በቆዳዎ ቁስለት (ቁስሎች) ምክንያት በግብረ *ሥጋ* ግንኙነት ጊዜ ምን ያህል ጊዜ ችግር አጋጥሞዎት ያውቃል?	0	0	1	2	3	4
15.	እርሶ ወደ ጤና አገልግሎት ተቋም በሚመጡበት ግዜ የእርሶዎን የቤት እና የስራ ደርሻ ሸፍኖ ለሚሰራ ሰው ምን ያህል ጊዜ መክፈል ነበረብዎት?	0	0	1	2	3	4
16.	ሌሎች ሰዎች በቆዳ ቁስለት ላይ ባላቸው ጥሩ ያልሆነ አመለካከት ምክንያት የአለባበስ ዘይቤዎን ምን ኣህል ጊዜ ቀይረዋል? (ልብስ፤ ሻርፕ፣ መነፅር፣ ኮፍያ፣ ማስክ፣ …. ወዘተ)	0	0	1	2	3	4
17.	በቆዳ ሊሽማኒያሲስ *(ቁንጭር)* ምክንያት በማህበራዊ እንቅስቃሴዎች (ሰረግ፣ ተዝካር፣ ሞት፣ ቅሬ፣…ወዘተ) ላይ ከመሳተፍ ምን ያህል ጊዜ ተቆጥበዋል?	0	0	1	2	3	4
18.	የቆዳ ሊሽማኒያሲስን *(ቁንጭር)* ለመታከም ወደ ሕክምና ቀጠሮዎ በሚመጡበት ወቅት ምን ያህል የሌሎች ሰዎች እርዳታ ያስፈልጎታል?	0	0	1	2	3	4
	**ስለ ጤና አገልግሎቶች እና ህክምና ግንዛቤ**	ኣላውቅም	በጣም ጥሩ	*ጥሩ*	*ደህና*	*መጥፎ*	*በጣም መጥፎ*
19.	የቆዳ ሊሽማኒያሲስ *(ቁንጭር)*ን ለማከም ይወስዱት ስለነበረው መድሃኒት ምን ያስባሉ?	0	0	1	2	3	4
20.	የቆዳ ሊሽማኒያሲስ*(ቁንጭር)*ን **ምርመራ ፈልገዉ** ወደ ጤና አገልግሎት መስጫ ተቋማት በመጡበት ወቅት ስለተደረገሎት አቀባበል ምን ያስባሉ?	0	0	1	2	3	4
21.	የቆዳ ሊሽማኒያሲስ*(ቁንጭር)*ን **መድሃኒት ፈልገዉ** ወደ ጤና አገልግሎት መስጫ ተቋማት በመጡበት ወቅት ስለተደረገሎት አቀባበል ምን ያስባሉ?	0	0	1	2	3	4
		ኣይመለከተኝም	በጭራሽ	*ከስንት አንድ ግዜ*	አንዳንድ ጊዜ	ብዙ ጊዜ	በጣም ብዙ ጊዜ
22.	ከቆዳ ሊሽማኒያሲስ *(ቁንጭር)* ለመዳን በወሰዱት መድኃኒቶች ምክንያት ምን ያህል ጊዜ ህመም ተሰምቶዎታል?	0	0	1	2	3	4
23.	የህክምና እቃዎችን እንዲቀርቡልዎት ወይም የቁስሉን ማሸጊያ እንዲለወጥሎት ምን ያህል ጊዜ በህክምና አገልግሎቶች ላይ ተማምነዋል?	0	0	1	2	3	4
		ኣይመለከተኝም	*ምንም*	*በጥቂቱ*	በመጠኑ	መሀከለኛ	ከፍተኛ
24.	የቆዳ ሊሽማኒያሲስ *(ቁንጭር)* ህክምናን ለማግኘት የሚደረግ ጥረት ምን ያህል አስፈላጊ ነው ብለው ያስባሉ?	0	0	1	2	3	4
25.	የቆዳ ሊሽማኒያሲስ *(ቁንጭር)* ጋር በተያያዘ ምርመራዎችን ለማካሄድ ፣ የሕክምና ቀጠሮዎች ወይም ሆስፒታል ለመተኛት ምን ያህል ጊዜ ይወስዳል?	0	0	1	2	3	4
