# Supplementary material for: The Cutaneous leishmaniasis impact questionnaire: Translation, cross-cultural adaptation and validation in adults with Cutaneous leishmaniasis in Ethiopia
Source: PLoS Negl Trop Dis. 2026 Mar 13;20(3):e0014073. doi: 10.1371/journal.pntd.0014073 (PMC12987440; doi:10.1371/journal.pntd.0014073)
Supplement: Attachment — Submitted filename: PNTD-D-25-00642.docx [file pntd.0014073.s004.docx]

1. S Table I is in ampharic and unable to understand
2. S Table 2 is in ampharic and unable to understand
3. Line 325: What is the rationale of having two nursing officers to perform content validity of CLIQ
4. 224: Face validity has to be done using more than 10 individuals. Usually 15 (5 from one location x3). N=5 in this study may be insufficient. But this was identified as a limitation by the authors.
5. line 252: Classification of Physician determined severity of CL (mild, moderate, or severe) has to have guidelines as without a guideline, the interpretation could be quite subjective. However, they have used a previously published method.
6. line 256: No guidelines for “minor improvement”, “substantial improvement”, Could be very subjective.
7. Line 261: Inter-rater reliability has to be performed by 2 raters. Not one rater repeating after 60 minutes. That is Intra-rater reliability and that also needs a minimum of 24 hr gap to prevent recall memory.
8. Suggest to add the standard cutoff values of ICC scores for the reader to interpret easily. This can be added as foot notes under Table 2.
9. Include meaning of abbreviations as foot notes in tables including in supplementary tables
10. Include the significant levels (P values) in Fig 1 between day 0 and Day 90.
11. Overall this is quite a subjective assessment method. Need improvement by using definitions in future.
12. Discussion needs improvement and needs to describe the application and the usefulness of CLIQ
13. Statistical analysis methods are good.
14. Overall this study describes a very subjective assessment method. Need improvement by using definitions in future

i.e How to categorize mild, moderate severe in CL?

How to categorize, minor and moderate improvement etc.

15. Discussion needs improvement and needs to describe the application and the usefulness of CLIQ
16. CLIQ has already been validated by Galvão et al. in 2018 and the need to translate into a local language and validating it again is important to the local community.
17. I do not see the importance of ampharic version to the international community. Suggest to submit this paper to a local journal
