# Supplementary material for: The Cutaneous leishmaniasis impact questionnaire: Translation, cross-cultural adaptation and validation in adults with Cutaneous leishmaniasis in Ethiopia
Source: PLoS Negl Trop Dis. 2026 Mar 13;20(3):e0014073. doi: 10.1371/journal.pntd.0014073 (PMC12987440; doi:10.1371/journal.pntd.0014073)
Supplement: Attachment — Submitted filename: Point by point Response.docx [file pntd.0014073.s006.docx]

5^th^ November 2025

Re: PNTD-D-25-00642

“The Cutaneous Leishmaniasis Impact Questionnaire: translation, cross-cultural adaptation and validation in adults with Cutaneous Leishmaniasis in Ethiopia

Dear Sir/Madam

I am resubmitting the revised version of our manuscript (with and without track changes).

We are grateful for your and the reviewers’ time and constructive suggestions in shaping our manuscript. We have modified the manuscript as relevant and included.

Below are the point-by-point responses to all the comments.

Yours sincerely,

Derese Bekele Daba on behalf of the authors

| **Comments to the Authors** | **Response from Authors** |
| --- | --- |
| **Editor** | |
| 1. Please upload the main figure as a separate Figure file in .tif or .eps format. For more information about how to convert and format your figure files please see our guidelines: | - We have submitted the files in the relevant format. |
| 2. We have noticed that you have uploaded Supporting Information files, but you have not included a complete list of legends. Please add a full list of legends for your Supporting Information file (CLIQ_Amharic version.pdf) after the references list. Please ensure that the supplementary tables are labeled correctly. | - We have added legends for the Supporting Information files after the reference list |
| 3. Please amend your detailed Financial Disclosure statement. This is published with the article. It must therefore be completed in full sentences and contain the exact wording you wish to be published. State the initials, alongside each funding source, of each author to receive each grant. For example: "This work was supported by the National Institutes of Health (####### to AM; ###### to CJ) and the National Science Foundation (###### to AM)." | - The detailed financial support is disclosed as follow in the main manuscript “The Skin Health Africa Research Programme is funded by the Research and Innovation for Global Health Transformation (RIGHT) Programme (Grant Reference Number NIHR200125 – awarded to SML, CP, MM, SLW, EG) of the National Institute for Health and Care Research (NIHR) https://www.nihr.ac.uk/. The funder had no role in study design, data collection and analysis, decision to publish, or preparation of the manuscript.” |
| 4. Your current Financial Disclosure states, " The Skin Health Africa Research Programme (SHARP) is funded by the Research and Innovation for Global Health Transformation (RIGHT) Programme [Grant Reference Number NIHR200125] of the National Institute for Health and Care Research (NIHR) https://www.nihr.ac.uk/. The funder had no role in study design, data collection and analysis, decision to publish, or preparation of the manuscript. ".   However, your funding information on the submission form doesn't indicate any funds. Please ensure that the funders and grant numbers match between the Financial Disclosure field and the Funding Information tab in your submission form. Note that the funders must be provided in the same order in both places as well. | - This change has been made during re-submission |
| If any authors received a salary from any of your funders, please state which authors and which funders. | - SML, CP, SLW and MM were partly funded through RIGHT Programme Grant NIHR200125. |
| **Reviewer: 1** | |
| **Method** |  |
| 1. Line 325: What is the rationale of having two nursing officers to perform content validity of CLIQ | - Thank you for your comment; the inclusion of two nursing professionals in the content validity assessment was deliberate. The Nurses work in the dermatology OPD or/and Ward, are often the primary point of contact for patients with dermatology conditions, playing a central role in patient counseling, wound care, treatment follow-up, and health education. Their practical experience and close interaction with patients provided valuable input into the clarity, cultural appropriateness, and relevance of the questionnaire items from a patient care perspective. Including experts from different disciplines ensured that the content validity process captured multidisciplinary viewpoints and enhanced the comprehensiveness and applicability of the instrument. |
| **Result** |  |
| 2. S Table I is in ampharic and unable to understand 3. S Table 2 is in ampharic and unable to understand | - Thank you for pointing this out. We appreciate your observations. The table S Table I (previously in Amharic) is now in both English and Amharic to ensure clarity and facilitate understanding for all readers. |
| 4. Line 224: Face validity has to be done using more than 10 individuals. Usually 15 (5 from one location x3). N=5 in this study may be insufficient. But this was identified as a limitation by the authors. | - Thank you for the comment; We agree that a larger sample would have been preferable for face validity testing. This limitation has been clearly acknowledged under discussion section from line 328-330. |
| 5. line 252: Classification of Physician determined severity of CL (mild, moderate, or severe) has to have guidelines as without a guideline, the interpretation could be very subjective. However, they have used a previously published method. | - To minimize subjectivity in classifying CL severity, we are based on the clinical judgment of dermatologists using criteria such as number, size, and site of lesions, and presence of complications, etc. - From our previously study inter-reliability study involving 12 clinicians who independently assessed lesion morphology, size, and severity in 12 patients with parasitologically confirmed CL. These findings informed a standardized approach for classifying severity (mild, moderate, severe) in the current study. We have added the reference for the study, and our published protocol. - And we have addressed the subjectivity of this measure in the Limitations section of the manuscript. |
| 6. line 256: No guidelines for “minor improvement”, “substantial improvement”, Could be very subjective. | - We have added a sentence to clarify this (page 6, line 160-164. In this study, we employed a standardized operational definition to classify treatment outcomes. A *minor improvement* was defined as flattening of 1–50% of lesions, a *substantial improvement* as flattening of 51–99% of lesions, and *cure* as complete (100%) lesion resolution or full re-epithelialization. These definitions were consistently applied and strictly adhered to by all study physicians throughout the study period. |
| 7. Line 261: Inter-rater reliability has to be performed by 2 raters. Not one rater repeating after 60 minutes. That is Intra-rater reliability and that also needs a minimum of 24 hr gap to prevent recall memory. | - We confirm that inter-rater reliability was performed: each participant was interviewed twice by two different interviewers. We have revised the methods for clarity. - We Updated the Methods section (page 7, line 168-172) |
| 8. Suggest to add the standard cutoff values of ICC scores for the reader to interpret easily. This can be added as foot notes under Table 2. | - Thank you for the suggestion. We have added the standard ICC cutoff values in the Methods section on line number 171 & 172 to aid interpretation. Table 2 presents the internal-consistency reliability of the CLIQ. (page 11, line 245-250) |
| 9. Include meaning of abbreviations as foot notes in tables including in supplementary tables | - We have added footnotes in tables. See page 10, line 236-237, and Page 11, line number 246-247. |
| 10. Include the significant levels (P values) in Fig 1 between day 0 and Day 90. | - We have addressed this in the paragraphs from line 276- 281. We developed the plot with P-value embedded on top of each category which we finally realized as affecting the attractiveness of the plot. So that we preferred to express the significance through narration. |
| **Conclusion** |  |
| 11. Are the limitations of analysis clearly described?   **yes, but not in a very acceptable manner**. | - Clarified and consolidated all key study limitations in the Discussion section. (Page 15, line 327-332. |
| 12. Do the authors discuss how these data can be helpful to advance our understanding of the topic under study?   **not in a satisfactory manner** | - Expanded the Discussion section to explicitly describe the significance and potential applications of the study findings. Please see line number 337- 344 |
| 13. Is public health relevance addressed? **limited** | - Yes, we believed that the last paragraph of the Introduction section addresses how relevant the translated and validated Amharic version will be to Ethiopians specially for those who are from endemic areas as well as for clinicians. Furthermore, the last paragraph of Discussion section also addressed CLIQ’s importance in Ethiopian context. |
| **Summary and General Comments** |  |
| 14. Overall this study describes a very subjective assessment method. Need improvement by using definitions in future i.e How to categorize mild, moderate severe in CL? | - To minimize subjectivity in classifying CL severity, we are based on the clinical judgment of dermatologists using criteria such as number, size, and site of lesions, and presence of complications, etc. - From our previous inter-reliability study involving 12 clinicians who independently assessed lesion morphology, size, and severity in 12 patients with parasitologically confirmed CL. These findings informed a standardized approach for classifying severity (mild, moderate, severe) in the current study. We have added the reference for the study, and our published protocol. - And we have addressed the subjectivity of this measure in the Limitations section of the manuscript. |
| 15. How to categorise, minor and moderate improvement etc.? | - We have clarified the criteria for this. “Responsiveness to change of the CLIQ following treatment was assessed by comparing CLIQ scores at Day 1 and Day 90 in individuals classified using physician global assessment as worse/no change, minor improvement (1–50% of the lesion flattened), substantial improvement (51–99% flattened or re-epepithelialized) and cure (complete flattening or re-epithelialisation of the lesion) which is stated on page 6 line number 160-164. |
| 16. Discussion needs improvement and needs to describe the application and the usefulness of CLIQ | - We have improved the discussion section through adding a summary of findings comparing the current result with previous findings as well as through adding relevance of translating and validating CLIQ in Amharic language which is found on page 14, line number 305-319. |
| 17. CLIQ has already been validated by Galvão et al. in 2018 and the need to translate into a local language and validating it again is important to the local community. | - Thank you |
| 18. I do not see the importance of ampharic version to the international community. Suggest to submit this paper to a local journal | - Ethiopia has one of the highest burdens of CL in Africa, as such tools that are suitable for use in Ethiopia are of public health importance for the global health control of CL. - Demonstrating the validity of the CLIQ settings other than Brazil where it was developed lends creditability to the tool which is of importance to the global CL community. - While the Amharic version of the CLIQ is specifically adapted for Ethiopian patients, it also provides important insights for the international community. The study demonstrates the process of cross-cultural adaptation and validation of PROMs in CL-endemic settings, which can serve as a model for other regions and languages. Additionally, our findings contribute to global efforts to standardize HRQoL assessment in CL and highlight the relevance of culturally appropriate tools in clinical trials and public health programs. |
| **Reviewer: 2** | |
| 19. This is a diligently conducted study. I wondered why the range of scores is so wide in each of the pre- and post treatment category. It is the experience with the original CLIQ and DLQI questionnaires as well? | - It’s because of the inherent nature of the tool. As it can be understood from the English version of the tool, most of items under post treatment categories are related to treatment and service satisfaction in which the scores would be null or minimal for those who had no treatment experience so far. This was already noticed by the developing authors in another study (. https://doi. Org/10.1371/journal.pone.0334215) - Therefore, the score of Cluster 1 and 2 for Day 1 varied but at Day 90 it became narrower |
| 20. Are any component of the Amharic CLIQ contributing to this noise? | - This is similar with the above comment. We didn’t notice an item making noise to the clusters rather it was because of the inherent nature of the tool. |
| 21. How different are the “somewhat better” compared to those who were“somewhat worse", can CLIQ MCID actually be recommended in other languages? | - The “somewhat better” and “somewhat worse” categories were based on participants’ self-reported opinions using a Likert scale. In our sample, very few participants rated themselves as “somewhat worse,” which limited meaningful comparison between these groups. Therefore, while the estimated MCID provides an indication of clinically meaningful change for the Amharic version, we recommend that the MCID be re-evaluated and validated separately in other language versions to account for potential cultural and linguistic differences in patient perception. - Clarified in the Discussion sections as limitation. (See Page 14-15, line 323-332) |
| 22. I think the discussion could be a bit more meaty with comparison with CLIQ adaptations in other contexts | - Thank you for the feedback. We have now amended the discussion making it deeper in analyzing the current findings and comparing them with similar previous studies. |
| **Reviewer: 3** | |
| **Method** | |
| 23. The names of certain statistical test and other important terms have been misspelled these should be corrected throughout the manuscript. | - We have re-checked the main manuscript and aligned statistical tests with respect to the intention and assumption followed. |
| 24. The Face Validity section mentions that a modification was made based on interview results but does not specify what modification was made. | - We have included the major changes after face validity under line from 209 – 217 of page 8. This is also clearly reflected in the tool. |
| 25. In the Validity Assessment section, “Known-group construct validity was assessed with physician-determined severity of CL (mild, moderate, or severe) and clinical phenotype: LCL, MCL, and (20, 23).” The phrase ends abruptly with “and” and an incomplete list, missing “DCL.” Complete the list by adding “DCL”. | - Thank you for pointing this out. We have completed the list by adding “DCL” |
| 26. The term global physician assessment (GPA) is introduced in the "Socio-demographic and Clinical Data" section without defining it earlier in the text. The author should mention the definition of GPA in the manuscript. | - Added a definition of GPA in the Methods section under “Data collection tools, procedures, and analysis.” (see page 5, line 139-142. |
| 27. The 'Data collection tools, procedures, and analysis' section mentions global model fit indices (e.g., χ²/df < 5.0, RMSEA ≤ 0.08) but does not clarify whether these thresholds were met in this study. The authors should state whether the model met these criteria. | - This was mentioned under line number 257- 266 under the result part on page 12 |
| **Result** | |
| 28. Throughout the results section of the main manuscript, the median IQR are presented as single value. However, the IQR should be reported as a range (Q1 to Q3). This needs to be clarified. | - Thank you for the comments. We have now changed single value of IQR to Q1 and Q3 |
| 29. Table 1: The table reports data for “Global physician assessment at day 90 (N=115)” and “Participant Opinion at Day 90 (N=115),” indicating that only 115 of the 250 participants were assessed at Day 90. However, the text does not explain why only 115 participants were included. | - We have added the reason for reduced sample size at D90 on page 6 line number 153 & 154. |
| 30. Table 1: “Number of Lesion” should be pluralized to “Number of Lesions,” and “Total size of the lesion” should be “Total Size of Lesions” for consistency with multiple lesions. | - Updated Table 1as suggested |
| 31. Table 1: The table lists “Total size of the lesion” with a cutoff of “Less than 40 mm” and “40 mm or more,” but it’s unclear whether this refers to the diameter of a single lesion or the cumulative size of multiple lesions. | - As the measurement refers to the diameter of a single lesion, we have rephrased the heading to “Size of the Largest Lesion” for clarity. Page 9-10, table 1 |
| 32. Reliability assessment: The Cronbach’s alpha for Cluster 2 (0.415) is notably low, which is below the commonly accepted threshold for adequate internal consistency (≥0.7). The authors should address the low Cronbach’s alpha value for Cluster 2. | - Thank you for the feedback . We have noticed this reduced level of reliability assessment among items under Cluster 2, which is attributable to the nature of the items rather than lack of consistency. All Items under Cluster 2 were supposed to be asked after certain treatment experience but in this case the items were asked at first date of visit leading to positive response only from those who had prior treatment experience. Further analysis of items under cluster 2 also showed Item Cluster total correlation >0.3 as well as no significant change to overall cronbach Alpha if Item deleted section. Therefore, rather than removing items due to low cronbach Alpha, we preferred to keep and check for other parameters. |
| 33. Reliability assessment: The number of items in Cluster 1 and Cluster 2 are not specified in the main manuscript and should be provided for ease of interpretation. | - 17 Items make cluster 1 whereas 8 items make Cluster 2 with over all items of 25. This is mentioned under instrunment section from line number 107-111, page 5 . |
| 34. Reliability assessment: The text does not specify whether the item-total correlations and Cronbach’s alpha values were calculated for the entire sample (n=250) or a subset, nor does it indicate the number of items in each cluster, which could affect interpretation of the alpha values. | - Dear reviewer, as indicated above, 17 items created Cluster 1 whereas 8 items go to Cluster II. Furthermore, the item-total correlations and Cronbach’s alpha values were calculated for the entire sample (n=250) in which the table showed n-250 just to simplify understandings. |
| 35. Table 2: Ensure all numerical values in this column have the same precision (e.g., two decimal places). For instance, PF6 has "1.40±1.60", which is consistent, but visually confirm all others align (e.g., TI22: "3.62±.89" should be "3.62 ± 0.89" for uniformity) | - Thank you for your suggestions. We have tried to express Mean and SD values with 2 decimal points whereas Item total correlation and cronbach alpha values with 3 decimal points. |
| 36. Table 2: Cluster 2 items HSS23 (0.096), TI21 (0.141), TI22 (0.108), HSS25 (0.117), and HSS26 (0.172) show low item-total correlations, suggesting weak construct alignment. The author should revise these items and further validate them to enhance the CLIQ tool’s convergent validity and utility. | We thank the reviewer for the thoughtful observation regarding the low item–total correlations for Cluster 2 items (HSS23, TI21, TI22, HSS25, HSS26). We agree that the observed correlations (<0.30) suggest weaker alignment with the total scale when measured at Day 1 (baseline). However, we would like to clarify that these items were designed to capture treatment-related experiences and perceived changes that typically emerge **only after undergoing treatment or follow-up clinical consultations**.  Specifically, the items in this cluster focus on aspects such as:   - Satisfaction with treatment experience - Treatment outcomes or improvement - Changes in functional limitation - Trust and interaction with health service providers   At Day 1 (D1), many participants had **not yet initiated treatment** or had **insufficient exposure** to clinical care to meaningfully respond to such items. Consequently, their responses were expected to be either neutral or non-differentiating at this timepoint, resulting in lower item–total correlations when analyzed together with baseline items.  This pattern does **not necessarily indicate weak construct validity**, but rather reflects **the temporal nature of the construct** being measured |

Thank You!
