# Supplementary material for: The Cutaneous leishmaniasis impact questionnaire: Translation, cross-cultural adaptation and validation in adults with Cutaneous leishmaniasis in Ethiopia
Source: PLoS Negl Trop Dis. 2026 Mar 13;20(3):e0014073. doi: 10.1371/journal.pntd.0014073 (PMC12987440; doi:10.1371/journal.pntd.0014073)
Supplement: Attachment — Submitted filename: point_by_point_response_auresp_2.docx [file pntd.0014073.s007.docx]

**Point by point response to Reviewers' comments:**

**Methods**


-Are the objectives of the study clearly articulated with a clear testable hypothesis stated?

-Is the study design appropriate to address the stated objectives?

-Is the population clearly described and appropriate for the hypothesis being tested?

-Is the sample size sufficient to ensure adequate power to address the hypothesis being tested?

-Were correct statistical analysis used to support conclusions?

-Are there concerns about ethical or regulatory requirements being met?

Reviewer #4: This paper clearly describes in detail the process to translate, culturally adapt and validate CLIQ scale to be used in Ethiopia.

The study methodology is complete. Just to clarify, kindly suggest adding a few additional details:

Comment 1)Line 207: Please include what are CLIQ domains Scale

**Feedback**:The CLIQ has seven domains and two clusters. This was mentioned in the methods section from lines 107- 113.

Comment 2)Line 210: Please clarify if "Higher scores indicate greater impact" means worst evaluation?

**Feedback**: These was accepted and added in the result section from line 210-213. We actually reported this in the methods section, too.

Comment 3)Line 214: Please include what are the DLQI domains Scale

Feedback: The above mentioned information were included under methods section from line number 114-120

To facilitate text flow and comprehension:

Comment 4) Line 252. Section: Validity Assessment: Include and describe subsections: Face validity, Content validity, Construct validity and Criteria Validity

**Feedback**: Dear reviewer thank you for the suggestion. We deliberately formatted the flow of translation and some validation contents (Face and content validity) as indicated in the manuscript. These is because of the fact that translation processes were supported by Face and content validity. As indicated in the methods section, following forward and backward translation, a group of patients were invited to participate in an interview to identify suitability of the questions followed by professional scoring for relevance of each of the items to measure each of the domains. These process contains few number of patients and professionals before implemented in large segment of patients.

The structures suggested is more of logical but we arranged following its technical flow.

Comment 5) Line 267. Section: Data collection tools, procedures, and analysis: kindly suggest organize the statistical analysis by Validity and Reliability evaluation

**Feedback**: This comment is accepted and we modified data collection tools, procedures and analysis plans as per technical flow of Validity and reliability assessment except for Face and content validity which we intentionally made them following back ward and forward translation. This creates technical flow rather than the usual theoretical flow.

**Results**


-Does the analysis presented match the analysis plan?

-Are the results clearly and completely presented?

-Are the figures (Tables, Images) of sufficient quality for clarity?

Comment 6: Results are presented according with the analysis plan.
Following the previous recomendations, and to be consistent, the authors can decide if the results could be presented in the same order: Validity Assessment: Face, Content, Construct, Criteria Validity, and Reliability Assessment.

Feedback: As described above, due to technical considerations rather than theoretical sequencing, we prefer to retain the current presentation of the validity and reliability assessments as outlined in the Methods and Results sections.

**Conclusions**


-Are the conclusions supported by the data presented?

-Are the limitations of analysis clearly described?

-Do the authors discuss how these data can be helpful to advance our understanding of the topic under study?

-Is public health relevance addressed?

Comment 7: The conclusions are clear, and study limitations are presented.
Will be good to explain more details about why the CLIQ is not correlated with the DLQI scale. In addition, to explain why cluster 2 had less internal consistence and less AVE compared with cluster 1.

**Feedback**: In general, we observed that Cluster 2 of CLIQ tool exhibited lower but significant measurements in majority of validity assessments. These is because of the fact that items in cluster 2 are appropriate and should be asked for someone who has prior exposure to treatment (either local or modern) at Day 1 assessment. If not, their response to each of items in Cluster 2 (19-25 items in the tool) would be “I don’t know or NA”, making the score very low.

The weak correlation observed between CLIQ and DLQI is expected, as CLIQ is a disease-specific instrument designed to capture psycho-social and stigma-related impacts unique to cutaneous leishmaniasis, which are not fully addressed by the generic DLQI. This finding supports the discriminant validity and added value of CLIQ.

**Editorial and Data Presentation Modifications?**


Use this section for editorial suggestions as well as relatively minor modifications of existing data that would enhance clarity. If the only modifications needed are minor and/or editorial, you may wish to recommend “Minor Revision” or “Accept”.

Comment 8: Please, review the titles and labels of supplementary tables.

**Summary and General Comments**


Use this section to provide overall comments, discuss strengths/weaknesses of the study, novelty, significance, general execution and scholarship. You may also include additional comments for the author, including concerns about dual publication, research ethics, or publication ethics. If requesting major revision, please articulate the new experiments that are needed.

Reviewer #4: This study is relevant to evaluate the Health-Related Quality of Life (HRQoL) of ´patients with cutaneous leishmaniasis in Ethiopia.

PLOS authors have the option to publish the peer review history of their article ([what does this mean?](https://track.editorialmanager.com/CL0/https://journals.plos.org/plosntds/s/editorial-and-peer-review-process%23loc-peer-review-history/1/010f019c03ab279d-e821a087-8abc-4101-abe1-fa843078a609-000000/Dbr6O4IflQypQBQTMGk2nzmmjVfO4dWdEinf7Q1qDS4=247)). If published, this will include your full peer review and any attached files.


If you choose “no”, your identity will remain anonymous but your review may still be made public.


**Do you want your identity to be public for this peer review?** For information about this choice, including consent withdrawal, please see our [Privacy Policy](https://track.editorialmanager.com/CL0/https://www.plos.org/privacy-policy/1/010f019c03ab279d-e821a087-8abc-4101-abe1-fa843078a609-000000/85B7b_bYYI29bERSXTUaOMq7eTnUUy9aYIpaBmIzRxI=247).

Reviewer #2: No

Reviewer #4: No

[NOTE: If reviewer comments were submitted as an attachment file, they will be attached to this email and accessible via the submission site. Please log into your account, locate the manuscript record, and check for the action link "View Attachments". If this link does not appear, there are no attachment files.]

**Figure resubmission:**

While revising your submission, we strongly recommend that you use PLOS’s NAAS tool ([https://ngplosjournals.pagemajik.ai/artanalysis](https://ngplosjournals.pagemajik.ai/artanalysis)) to test your figure files. NAAS can convert your figure files to the TIFF file type and meet basic requirements (such as print size, resolution), or provide you with a report on issues that do not meet our requirements and that NAAS cannot fix.

After uploading your figures to PLOS’s NAAS tool - [https://ngplosjournals.pagemajik.ai/artanalysis](https://ngplosjournals.pagemajik.ai/artanalysis), NAAS will process the files provided and display the results in the "Uploaded Files" section of the page as the processing is complete. If the uploaded figures meet our requirements (or NAAS is able to fix the files to meet our requirements), the figure will be marked as "fixed" above. If NAAS is unable to fix the files, a red "failed" label will appear above. When NAAS has confirmed that the figure files meet our requirements, please download the file via the download option, and include these NAAS processed figure files when submitting your revised manuscript.

**Feedback**: Figure 1 was uploaded to the suggested website and TIF file format was downloaded after authomatic adjustment.

**Reproducibility:**

To enhance the reproducibility of your results, we recommend that authors of applicable studies deposit laboratory protocols in [protocols.io](http://protocols.io/), where a protocol can be assigned its own identifier (DOI) such that it can be cited independently in the future. Additionally, PLOS ONE offers an option to publish peer-reviewed clinical study protocols. Read more information on sharing protocols at [https://plos.org/protocols?utm_medium=editorial-email&utm_source=authorletters&utm_campaign=protocols](https://plos.org/protocols?utm_medium=editorial-email&utm_source=authorletters&utm_campaign=protocols)
